# Attitudes to ageing amongst health care professionals: a qualitative systematic review

**DOI:** 10.1007/s41999-023-00841-7

**Published:** 2023-08-08

**Authors:** Neil Jeyasingam, Loyola McLean, Lisa Mitchell, Anne P. F. Wand

**Affiliations:** 1 Specialty of Psychiatry, Faculty of Medicine and Health, The University of Sydney, Chippendale, NSW 2006 Australia; 2 Mental Health Drug and Alcohol, Far West Local Health District, Broken Hill, NSW Australia; 3 Brain and Mind Centre, Faculty of Medicine and Health, The University of Sydney, Sydney, Australia; 4 Westmead Psychotherapy Program for Complex Traumatic Disorders, Cumberland Hospital, WSLHD, North Parramatta, Australia; 5 Barwon Health, Geelong, VIC Australia; 6 Discipline of Psychiatry and Mental Health, Faculty of Medicine and Heath, University of New South Wales, Sydney, Australia; 7 Older Peoples Mental Health, Sydney Local Health District, Sydney, Australia

**Keywords:** Ageism, Clinician, Perceptions, Burnout, System

## Abstract

**Objectives:**

Older adults utilising healthcare may be vulnerable to systemic bias regarding ageing potentially affecting care. Primary aim is to systematically review the qualitative literature examining attitudes to ageing amongst health care professionals (HCPs). Secondary aim is to describe and compare attitudes to ageing between different professional groups.

**Methods:**

Studies examining attitudes to ageing of HCPs were identified using four databases. Original qualitative research with HCPs was included, excluding studies examining students alone, reviews, and grey literature. Three authors undertook standardised data extraction and quality rating for each study and thematic synthesis, with a fourth senior author reviewing. Quality appraisal followed the Attree and Milton guidelines (2006), including studies rated A–C.

**Results:**

Of 5869 citations, 13 met initial inclusion criteria. Synthesis of the 12 studies graded A–C followed (excluding one study rated-D). Nurses, doctors, and social workers were most frequently investigated. Identified themes included Attitudes towards older persons, The role of the older persons’ family, Behaviour of HCPs towards older persons, Behaviour of older persons towards HCPs, and Definitions of an older person. An overarching theme emerged of the systemic context of attitudes to ageing. Common attitudes were found across disciplines, with doctors emphasising complexity and dependency and nurses the burden of care.

**Conclusions:**

HCP’s attitudes to ageing vary and are shaped by professional experiences and systemic factors. HCP attitudes may affect responses to and care of the older person. Therefore, interprofessional research, and systemic and service changes across disciplines are required to foster real and sustainable attitudinal change to support older peoples’ care.

## Key summary points

**Aim:**

The primary aim was to systematically review the qualitative literature examining attitudes to ageing amongst health care professionals.

**Findings:**

Emergent themes included the attitudes and behaviours of healthcare professionals towards older people, role of family, definitions of an older person, and behaviours of older adults towards healthcare professionals. An overarching theme was the systemic context of attitudes to ageing.

**Message:**

Attitudes to ageing of multidisciplinary healthcare professionals are shaped within complex healthcare systems necessitating interprofessional approaches and systemic change.

## Introduction

Older adults utilise health care systems more than younger adults, and may be vulnerable to systemic bias regarding expectations of old age [1], counteracted by healthcare professionals (HCPs) with positive views of ageing [2]. Ageism is a social construct of old age; depicting ageing and older people in a stereotypical and often negative way [3]. Ageism may be manifest through negative attitudes, the knowledge and values individuals or systems may hold, and their behaviours [1, 4, 5]. Ageism can affect the care of older adults through limitations in care provided, barriers to accessing services, and HCP reinforcing internalised ageism [3].

Prior reviews examining the attitudes to ageing of HCPs have identified a paucity of studies [3, 6, 7]. Results have been difficult to interpret as study aims and outcome measures differ, including disparate concepts such as implicit ageism [6]. Most reviews evaluating this question have used quantitative methods [7–9]. By comparison, qualitative methods involve an inductive approach, permitting identification of new knowledge regarding perspectives of ageing, with breadth and depth of ideas facilitating understanding of subjective experience and meaning [10]. We are not aware of any qualitative literature reviews examining this topic.

The primary aim was to systematically review the qualitative literature examining the attitudes of HCPs to ageing. The secondary aim was to describe and compare attitudes to ageing between different professional groups.

## Methods

### Search strategy

PRISMA reporting guidelines were used to conduct this systematic review [11]. Searches were undertaken of four databases CINAHL, MEDLINE, PsycINFO, and EMBASE from June 1 2011 to January 1 2022 for studies exploring the attitudes of HCP to ageing using the following terms in AND/OR combinations:

Health Personnel, Physician, Medical Officer, Doctor, Medical Practitioner, Audiologist, Occupational Therapist, Speech Therapist, Physical Therapist, Physiotherapist, Psychologist, Nurse, Dentist, Social Worker, Healthcare professional, Allied Health Personnel, Home Health Aide, Community Health Worker, Surgeon OR Clinician AND Attitude, Attitude of Health Personnel, Attitudes towards ageing, Attitudes towards ageing AND/OR Attitudes about older patient AND Aging, Ageing, Ageism OR Age discrimination.

### Inclusion and exclusion criteria

Eligible studies for inclusion described original qualitative research with fully qualified HCPs were peer-reviewed and in English. Case reports/case series and the qualitative component of mixed methods studies were eligible for inclusion if reported in sufficient detail for data extraction.

Studies were excluded if participants were students or studies were from the grey literature, reviews, letters, editorials, commentaries, and conference abstracts for which data requests were unsuccessful. Studies that specifically pertained to implicit ageism were excluded, as they did not consider attitudes to ageing more broadly.

### Assessment of quality

Each study was appraised for quality using the Attree and Milton checklist for qualitative systematic reviews [12]. This checklist comprises nine quality, methodological, and ethical categories. For each category, a rating from A-D is determined; A: no or few flaws, to D: significant flaws threatening the validity of the study. Studies rated-D were excluded, as per the quality rating guide [12]. An overall score for each study is based on an average of scores in the nine checklist categories. Following independent rating by three reviewers (NJ, LiM, and AW), the reviewer scores were compared. A senior author (LM) reviewed the final quality ratings. For disagreements between the overall scores for each study, the reviewer scores for each checklist item were compared and discussed to reach consensus.

### Data extraction and synthesis

Three authors (NJ, LiM, and AW) conducted independent searches of the databases to identify articles eligible for review. Titles and abstracts were independently screened by three reviewers against inclusion and exclusion criteria. Disagreements were resolved via discussion to reach consensus. Full texts of identified abstracts were obtained and independently reviewed by the three authors for eligibility. The final articles were subject to standardised data extraction (Participants, Design and Methodology, Emergent Themes, and Quality Appraisal) by the three reviewers, with a fourth senior author (LM) reviewing for consensus. Reference lists of included articles were hand-searched to identify additional papers.

Thematic synthesis of included papers was undertaken using the method described by Thomas and Harden [13]. Three authors separately performed a line-by-line analysis of the Results section (direct quotes and description of emergent themes) in each paper, with each line coded for meaning and content. This initial step of thematic synthesis involved translating concepts from individual primary studies to the novel systematic review. As the results of each study were coded, categories were reviewed and refined to check for consistency of interpretation, and whether additional levels of coding were required. Similarities and differences between codes were examined to group them into a hierarchical structure. In this way, descriptive themes emerged from the inductive analysis of individual study findings. These descriptive themes were then examined in the context of the review questions to generate more abstract or analytical themes via formulated meanings. The individual author’s thematic syntheses were then compared and discussed until consensus reached. A fourth author reviewed the analysis to further appraise the final emergent themes. To stay close to the original empirical data, direct quotations were used to illustrate emergent themes. A secondary analysis of themes was performed to explore differences in themes between the professional groups.

### Reflexivity

Two authors are aged-care psychiatrists (NJ, AW); one is a geriatrician (LiM) and the fourth is a consultation-liaison psychiatrist and psychotherapist (LM). The clinical experience and guiding professional theories of the authors were considered during thematic synthesis and discussion (person-centred care, humanism, attachment, and trauma).

## Results

**Fig. 1:**
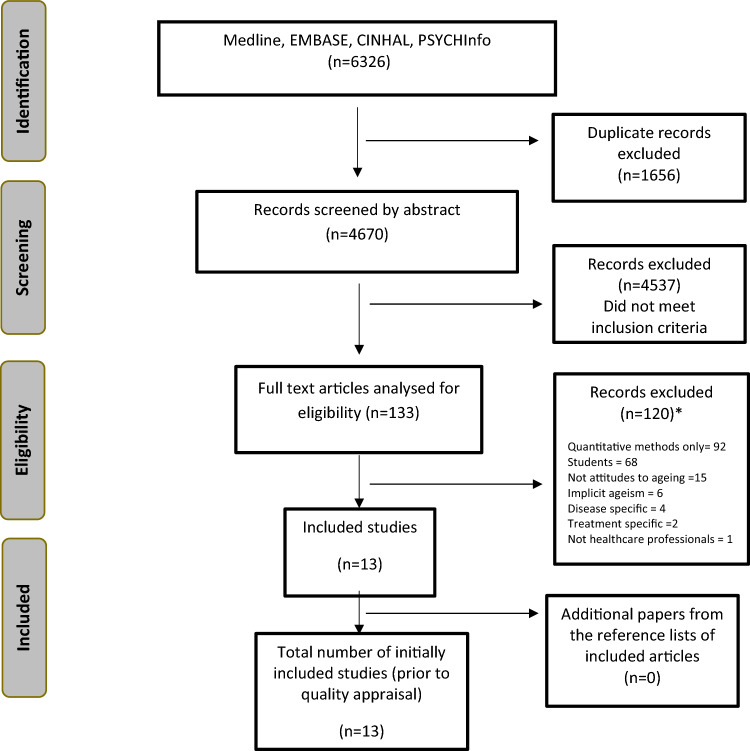
PRISMA flowchart of study selection. *Note that some studies were excluded for multiple reasons

Of 5869 citations identified, 13 met initial inclusion criteria [2, 14–25] (Fig. 1). One study was subsequently excluded due to a quality rating of D [25], leaving 12 studies for synthesis. HCPs studied included nurses, social workers, physicians, emergency department staff, psychiatrists, general practitioners, psychologists, an occupational therapist, and geriatric community health workers. Nine studies were rated A for quality, one B and two C.

The findings of the review and quality appraisal are shown in Table 1.

**Table 1:** Characteristics of reviewed studies

Study, country, and underlying qualitative theory (where described)	Setting and participants	Aim, study design	Summary of emergent themes	Quality rating	Strengths	Limitations
Ben-Harush et al. (2017) Israel	Medical and mental health professionals in long term care, primary care, and hospitals 29 clinicians: Physicians (16 F, 4 M), nurses (5 F) and social workers (4 F) Age not stated	Aim: to evaluate and compare ageism amongst physicians, nurses, and social workers Design: three focus groups were provided with 11 set questions	Perceived difficulties related to working with older adults and their families Invisibility of older people and discriminatory communication patterns with older patients (exclusion, disempowerment, patronising behaviour) Providing inappropriate care to older patients (fostering dependency to save time, aged-based treatment decisions)	A	Interviewer trained in qualitative research Clear statement of research aims Triangulation of responses of 3 different healthcare professional groups Method of thematic analysis well described Measures of trustworthiness reported (> 1 coder, peer debriefing, triangulation)	Limited scope for broader reflection with structured interviews Sample strategy of recruiting from clinicians attending education sessions may select those more motivated/ interested in older people Unclear if data saturation achieved
Bershtling et al. (2016) Israel Inductive approach	Hospital and community healthcare professionals 18: (8 Social workers and 10 Physicians) aged 28–58 and 15 older persons (aged 65–90)	Aim: to develop a better understanding of the right to health in old age Design: semi-structured focus group interviews	Self-positioning vis a vis the healthcare system (older adult patient perspectives) The Kafkaesque positioning of the health care system (referring to perceptions of the healthcare system, mainly those of older adults themselves: e.g., bureaucracy, lack of transparency and exclusion from care decisions limiting access to health rights) Social aspects influencing the right to health (healthcare professional views- system resources, family vs individualism, and a humane approach)	A	Utilised a separate observer to the moderator to take field notes and observe interactions Context well described Triangulation of responses of healthcare professional and older persons Clear description of content and form analysis Trustworthiness enhanced by having dual coders	Unclear how healthcare professionals were chosen or who declined to participate No comment on data saturation Results presented as a hybrid of theory, existing literature and derived themes, resulting in relatively less focus on empirical data and subjective meaning
Bulut et al. (2015) Turkey	Emergency Department 18 Emergency doctors and nurses Physicians (11) average age 30.78, Nurses (7) average age 28.23 years	Aim: assessment of the views of emergency service staff on ageing and older patients Design: mixed methods. Questionnaires* and focus group interviews with open-ended set questions *Quantitative results are not presented here	Understanding older patients’ situations (definitions of older adults) Good nursing care and medical treatment Factors affecting good nursing care (holistic, personalised) and medical treatment (optimising quality of life, diagnosis and safe treatment) Emotions experienced (by clinicians)—hopelessness, pity and stress	A	Clearly described aim and recruitment strategy, which captured half of the total possible sample in the qualitative arm Questionnaire data informed focus groups Triangulation between quantitative component and 2 different healthcare professional groups Robust method for data collection Implications for clinical practise and training were discussed	Method of thematic analysis not described Lacks consideration of reflexivity No separate analysis of qualitative data (quotations were used to support themes derived from questionnaire data) Unclear if data saturation was reached in qualitative analysis
Di Lorito et al. (2019) England Inductive approach	Forensic psychiatric secure services 13 participants: Psychiatrists (2), Specialty doctors (2), Specialist medical trainees (3), Nurses (4), Nursing assistants (2)	Aim: to explore views on how well secure services are meeting the challenges of an ageing population Design: focus groups with set topic guides	Identifying patient’s needs—protocols, staff skills and training, recognising cognitive impairment/dementia Addressing patient’s needs—facilitators (personalised care), barriers (lack of meaningful activity, invisibility, lack of resources) and service improvement (separation by age group, consumer input)	A	Researchers reflected on their potential biases and perspectives, demonstrating reflexivity Data collection and audit trail were well described. Triangulation of results with companion study of older patients in secure services Method of analysis well described Measures to optimise trustworthiness were described (e.g. negative cases)	No data on eligible participants declining to participate Data saturation not described No medium secure staff were recruited, although this is where the majority of older adults are
Craciun (2016) Romania Thematic analysis	General practitioners in public clinics or private practise 34 participants: 17 women, 17 men. All aged 30–60	Aim: to examine the views of general practitioners on old age and what role they perceive gender may have in their representations of ageing Design: individual episodic interviews with nine set questions regarding their experience with older adults and perceptions of ageing	De-Gendered representations of aging amongst GPs* (ageing as negative, chronological, subjective ageing and ageing well) De-gendered representations and actions towards older patients (difficult patients, dependent. vulnerable, not understanding, non-compliant) *The effect of gender was explored two ways: the gender of the GPs and whether GPs treat male or female older people differently	B	Thematic coding method well described Subjective meaning was privileged by use of frequent illustrative quotes Immersion in interview transcripts	Little detail provided on structured questions, particularly those relating to perceptions of gender (the key aim of the study) The method of data collection was not described (e.g. was audio-recording used?) Unclear if data saturation was achieved The analysis was not conducted in line with purported aims- e.g. data from male or female GPs were not thematically analysed as separate groups Data were not triangulated
Craciun and Flick (2016) Country not specified	Professionals working with older people or services pertaining to preparation for old age 7 participants: General Practitioner (1), psychologists (3), social worker (1), occupational therapist (1) and an Insurance agent (1)	Aim: to assess health care professional of multiple disciplines regarding their views on positive ageing Design: individual episodic interviews with set questions covering the participant’s work in services for ageing populations, representations of ageing, promotion of old age, and what factors interfered with their work	Negative representations of age (subtheme-ageing as decline) Patterns referring to the promotion of a positive old age (subthemes staying mentally fit, taking personal responsibility for positive ageing, being socially engaged, integrating technology in coping with ageing) Healthcare professional reflections on personal ageing (ability and activity rather than chronological age; attitude and adaptation to ageing)	A	Clear aims Systematic data collection Method of analysis well described Findings privilege subjective meaning (numerous illustrative quotes provided) Implications for clinical practise discussed	Context and setting of study are unclear Sampling strategy is not described Unclear whether data saturation was achieved Inadequate representation of healthcare professional disciplines Lacks consideration of reflexivity
Flatt et al. (2013) United States of America Grounded theory	Anti-ageing physicians and practitioners from an online directory 31 anti-ageing practitioners (71% medical doctors, 29% doctors of naturopathy, osteopathic medicine or nurse practitioner) Age 33–71 23 (74%) white/Caucasian 19 (61%) male	Aim: evaluate how descriptions of their work, definitions of ageing and goals for patients intersect with ‘successful ageing’ Design: semi-structured individual phone interviews	Personal responsibility for poor ageing (self-inflicted) Functional losses are not ‘normal’ ageing Ageing as a hormone deficit Loss of energy equated with ageing Ageing well is individually determined Good (less disease, less cost, productivity) vs bad ageing	A	Clear aims with corresponding study design well suited to investigate aims Measures taken for improving inter-rater reliability Data analysis well described Findings privilege subjective meaning (numerous illustrative quotes provided) Detailed discussion of factors influencing findings	Unclear whether data saturation achieved Reflexivity not considered Study limitations not described
Higashi et al. (2012) United States of America ethnography	General hospital 21 participants: interns, residents, medical students (numbers of each not stated)	Aims: explore attitudes of physicians in training to older patients Design: individual interviews and participant observation	Negative characteristics of old age (frailty, dementia, multiple chronic illnesses and lack of social support) Older patients inherently at end of life Cognitive impairment is assumed (older people are infantalised and perceived as frustrating) Therapeutic nihilism Complexity (both a plus and negative) Older people as time-consuming, needy, and inflexible Older people are better attended by other HCP Older people respect junior doctors	C	Robust data collection (recordings and observations) Triangulation of data sources Researchers immersed with participants in their clinical role	Sampling strategy not described Audit trail for observations not described No information on method of data analysis No comment on data saturation Reflexivity not discussed although researcher was embedded with medical team Authors did not consider limitations Poor consideration of implications for policy and practise No mention of ethical approval
Lee and Richardson, (2020) United States of America Inductive approach	Experienced (20 years +) geriatric community care workers from agencies providing home- and community-based services to older adults 20 participants: 85% were licenced social workers All women Aged 50–72	Aim: exploring the views of geriatric community health workers regarding retirement* and barriers/facilitators to ongoing engagement with the ageing population after retirement Design: Semistructured interviews *Results pertaining to views about retirement are not presented here	Facilitators: strong bonds to older adults (affection, compassion and passion to serve) Identifying themselves as a resource for older adults Barriers: Negative attitudes developed towards ageing and older adults Compassion fatigue	A	Clear aim and research question Method of thematic analysis is well described. Appropriate use of productive ageing framework to interpret data Trustworthiness enhanced by assessing inter-rater reliability of coders and peer review Implications for education and training discussed	Semistructured interview format not well described Limited variety of healthcare workers (85% licenced social workers) and all women Sample size not sufficient to achieve data saturation
Manasatchakun et al. (2018) Thailand Inductive, latent content analysis	Community nurses working in health promotion in Thailand 36 participants: aged 23–52 years One male participant	Aim: assess views of community nurses regarding healthy ageing Design: focus group interviews using prepared open-ended questions	Healthy ageing: -Being strong -Being a supporter and feeling supported Promoting healthy ageing: -providing health assessment -sharing knowledge -having limited resources (financial and workforce)	A	Data collection process clearly described (field notes, audio recordings, observations of nonverbal communication/power relations), all of which contribute to triangulation of interview and focus group data Clear audit trail of steps in thematic analysis Clear immersion in data Trustworthiness enhanced by dual coders and participant validation Reflexivity demonstrated Implications for policy discussed	Unclear whether saturation of themes achieved Funding not disclosed, which may be relevant as the first author is a nursing instructor in the region Sample included only those districts already involved in promotion of healthy ageing—other districts not sampled
Hosseini et al. (2020) Iran Content analysis	Educational and therapeutic hospitals Participants: nurses (13, 10 clinical, 3 ‘head’ nurses), educational supervisor (1) Work experience 4–21 years Age 25–52 5 women	Aim: elaborate the reasons for ageism at individual and system levels Design: semi-structured and in-depth individual interviews	Patient-related factors: older people are difficult, complex, dependent, cannot be helped Caregiver-related factors: therapeutic nihilism, older people should just be allowed to die Care provider system factors: waste of resources -Socioeconomic factors: self-inflicted problems Family related factors: lack of respect, absent	A	Aim well described Purposive sampling to maximise diversity of participants Data saturation achieved Data collection well described Method of analysis well described and results confirmed and validated Utilised respondent validation of results Considered study limitations	Limited application of findings to policy and practise Little consideration of reflexivity
Moore (2017) England	Nursing homes Participants: nursing home owners (12, 2 were nurses), nursing home managers (12, 10 were nurses), nurses/care staff (12)	Aim: unclear- relates to how personal values influence attitudes and behaviours towards residents Design: semi-structured face to face interviews	Older people are unworthy of communication Poor cognition ‘out of it’ Futility of treatment Proximity to death	C	Open questioning Implications of findings for policy development discussed	Study aims were unclear Sampling strategy not described No comment on data saturation Form of data collection not described, no mention of audit trail Method of data analysis not described Themes not clearly articulated Study limitations not considered Reflexivity not considered, although researcher was Commissioner of care and nursing home services No mention of ethical approval
Oeseburg et al. (2013) The Netherlands	6 GPs and 6 practise nurses	Aim: to develop and evaluate an interprofessional education programme for GPs and practise nurses (including examine knowledge and attitudes towards older people) Design: mixed methods (telephone interviews and questionnaire)	Elder care is more than just disease management Collaboration with other disciplines can change attitudes to elder care	D	Clear study aims Triangulation of qualitative and quantitative data	Context of the researchers was unclear, no reflexivity Unclear who gathered interview data No comment on data saturation No information on method of qualitative data analysis No quotations provided to illustrate themes No measures to enhance trustworthiness of findings No evidence provided to support change in attitudes of HCP to older people through education

### Thematic synthesis

Five themes emerged from the qualitative synthesis: Attitudes towards older persons, The role of the older persons’ family, Behaviour of HCPs towards older persons, Behaviour of older persons towards HCPs, and Definition of an older person. Illustrative quotations for each theme are presented in Table 2. An overarching theme was the systemic context of attitudes to ageing, encompassing resourcing shortfalls, burnout, and compassion fatigue (Table 3).

**Table 2:** Illustrative quotations for emergent themes

Theme subthemes	Illustrative quotation
Attitudes towards older persons	
(i) Rewarding and revered	“Working with the elderly is a very rewarding job.” (Nurse educator) [21] pp. 4
	“It’s the people, one of the things that I love about it is that… they have been around longer than I have. They’ve learned, and they’ve had this wealth of experience and information…. They all have something to offer.” (Geriatric Community Health Worker) [22] pp. 53
	“The aged should be admired by the community…” (Nurse) [23] pp. 61
	“To accept the loss of several abilities and interpret it not as loss, but rather as a change in your lifestyle … to realize it does not function as it used to, but think that there are alternatives, it can work in another way.” (Occupational therapist) [15] pp. 2877
(ii)Unwanted, dependent, and difficult	
(a) Unwanted and costly	“It is more pleasant to take care of a younger person. It is more aesthetic….Old age is not pretty…” (Nurse) [14] pp. 43
	“Having too many concomitant diseases in the same patient, prolonged treatment periods, the patient’s personality creating problems, failure to respond to treatment, and lacking the necessary treatment for a long time make a good medical treatment harder.” (Physician) [15] pp. 182
	“The first thing that comes to mind is…how to discharge or hospitalize the patient. I feel impatient.” (Physician) [15] pp. 183
	“…these old people don’t know what’s going on anyway. So they don’t know whether they’re clean or not, do they?” (Nurse) [24] pp. 4
	“They are like children. Most of them have Alzheimer’s disease or dementia… Older patients either do not understand or are unable to do as advised.” (Physician) [15] pp. 181
(b) Complex and challenging	“I felt really intimidated by older patients because they’re so complex and are usually on a whole bunch of drugs and have a lot of different medical problems.” (Trainee physician) [20] pp. 480
	“The elderly do not cooperate and their illnesses are complicated and care is also difficult and many are multifunctional…. Some colleagues sometimes say they do not like to take care of the elderly but others…. Saying things like ‘he is old, what can I do to him?’” (Nurse) [21] pp. 3
	“…working with elderly patients can get frustrating… often times there’s not a lot you can do.” (Intern) [20] pp. 479
	“….she is not willing to be helped at all. She can’t live by herself… She is so preoccupied with her rebellion that she won’t let anyone tell her what to do anymore. She won’t accept any help…” (Social worker) [14] pp. 43
Opposing pole: a welcome intellectual challenge	“I feel like I’ve had a really positive experience and am feeling more positive about geriatrics… dealing with their chronic illnesses and talking about what’s going to happen in the future, about falls risks, social supports, their home life- I just really enjoy that, and I feel more comfortable.” (Resident) [20] pp. 480
(c) Dependent	“a young person can take care of themself…. but the elderly… need a lot of care and all their daily tasks must be done for them.” (Nurse) [21] pp. 3
	“I always check how autonomous a person still is, how much does he or she depend on others for help.” (Psychologist) [17] pp. 2877
	“An older patient is a person over 65 years old, likely to require the assistance of either a health care or non-health care provider while performing everyday activities, a member of the most senior part of society, and requires more care following an illness, thereby causing an increase in the costs.” (Physician) [15] pp. 181
	“Young patients have enough strength to take care of themselves and to be assertive and self-assured with their physicians… this is not the case for older patients…. I will represent them and make sure they get the best possible treatment…” (Nurse) [14] pp. 43
Opposing pole: assertive and independent	“They are used to taking care of themselves.… the generation aging now is much more strong-minded.” (Occupational therapist) [17] pp. 2876
Role of the older persons’ family	
(a) Meeting expectations	“…the patient’s relatives who do not respect the nurses, in turn, make the nurses neglect the patient…. We see sometimes that the family of the patient themselves like their relative to die and are not bothered very much. It’s affecting our performance.” (Nurse) [21] pp. 4
(b) Abandonment	“They [the children] do not want to [help]; they have no time….. they don’t want to make the effort.” (Physician) [2] pp. 483 “…in the old days the family used to take responsibility and care for the old, but nowadays this is not the case anymore so old people need to be more independent.” (Occupational therapist) [17] pp. 2876
(c) Advocacy	“There is also difficulty even if they know their rights and the ways to implement them….. If they don’t have a son or a daughter accompanying them to help in realizing these rights, or if we do not take it upon ourselves to accompany them closely in realizing their rights, it is very difficult for them to take the initiative…” (Social worker) [2] pp. 483
Behaviour of healthcare professionals towards older persons	
(a) Dehumanising, discriminating and dismissing	“Sometimes people don’t talk much to older patients as they would a younger patient. People don’t explain as much to them, they kind of more just reassure and tell them they’re going to be OK… sometimes that’s totally app. ropriate… but at other times I think they want to know and …they could understand it and they’re not really given the chance.” (Intern) [20] pp. 478–9
	“Nurses can come and take out the catheter without even looking at or talking to the patient.” (Nurse) [14] pp. 44
	“…they [nursing carers] don’t speak to the old people… It’s like they aren’t worthy of communication.” (Nursing care staff) [24] pp. 3
	“Some colleagues who themselves have moral issues and may not prescribe the medication of elderly patients and keep that secret from everyone because it has consequences.” (Nurse) [21] pp. 3
	“One day the code was announced…. They said leave it, the patient is 90 years old, what else do you expect, leave them be…they delayed seeing to the patient and the patient died.” (Emergency worker) [21] pp. 3
	“I think there’s a certain amount of discrimination going on that those [older] patients may not get as good care as someone who comes in with a similar problem who’s 10–15 years younger, and can describe the problem well and establish a better rapport with the physician.” (Intern) [20] pp. 479
	“…older patients do not evoke the same attitude as younger patients… They [the older person] do not receive proper care in regard to how people talk to them or behave toward them.” (Physician) [2] pp. 483
Opposing pole: empowered role models	“We do not need to suggest or train them, just listen to them. “Phaya”, which is passed down from ancestors, can be used to apply health services to harmonize with the local context. The senior citizens have meetings to share what they do to stay healthy. We have an older person who is a health idol for the aged in other villages. We should support what older persons do.” (Community nurse) [23] pp. 62
(b) Unworthy of care	“Some of us see it all as a bit futile really so don’t bother. Futile because they [the nursing home residents] are going to die soon.” (Nurse) [24] pp. 4
	“Some staff say that what’s the point in spending time and money on this elderly patient? Of course the system, the family, and even the personnel do not say this publicly… they say it’s useless wasting energy on the elderly.” (Nursing educational supervisor) [21] pp. 4
	“…in the case of an elderly addict… he does not have mercy on himself, so why should I bother myself and care about him. If you don’t care about yourself and how old you are, why should we?” (Physician) [21] pp. 4
Behaviour of older persons towards healthcare professionals	
Challenging behaviours	"We are dealing with people who have behavior problems. They curse and hit.” (Nurse) [14] pp. 43
	“…the ones [older patients] who don’t pay attention and are rude, they use bad language, they display a superiority attitude, they threaten they will complain to the authorities.” (General practitioner) [16] pp. 5
	“Some [older] patients are completely disabled, but they want attention and are demanding…” (Doctor) [14] pp. 43
	“She just said yes to everything I told her, but she did not understand, in the end she did not know what she needed to do, so I had to talk to other child and explain all to him again” (General practitioner) [16] pp. 6
	“[compared to younger patients] They are less likely to complain, and nobody speaks for them, so they just don’t have a voice.” (Staff member low security unit) [18] pp. 278
Opposing pole: respect for clinicians	“…with older patients…. If you’re taking care of them then you’re the doc. They’ll listen to you.” (Intern) [20] pp. 480
Definition of an older person	
(a) More than chronological age	“There are people in their 80 s with all sorts of health problems… who are still active and others in their 70 s are very restricted in their actions… it is not a question of biological age anymore…” (Doctor) [17] pp. 2877
(b) Illness and dependency	“I think it should be more about ability than age. Cos the concept of age is very segregating whereas ability is more enabling. (Health Care Practitioner) [18] pp. 279
	“Old age is when you start depending on others, you start forgetting things, you cannot take care of yourself anymore, you forget your medicine, you have no appetite, and you start thinking about death and the life you had before.” (Doctor) [16] pp. 4
	"I believe you are old when your body is old, the illness is so bad that it cannot fight anymore and no medicine can help, not the age in years is relevant.” (Doctor) [16] pp. 4
	“Older patients are individuals who are 60–70 years old, on average, and have chronic diseases and complicated problems together with physical and mental distortions.” (Nurse) [15] pp. 181
	“…we know that when people are in a suboptimal state hormonally, their body drifts rapidly towards disease.” (Doctor) [19] pp. 950
	"Old is someone who is not healthy anymore and who experiences no joy in daily life… this makes one old… not having fun in life anymore… work and interests keep one young.” (Occupational therapist) [17] pp. 2875
	“Some of us see it all as a bit futile really so don’t bother. Futile because they [the residents] are going to die soon.” (Nurse) [24] pp. 4
Opposing pole: independent	“People with a good education level or a good job are normally more independent … those with poor education are more helpless …” (Social worker) [17] pp. 2876
(c) Taking responsibility for ageing	“Most of what they call age-related disease are things that come from lifestyle choices that have been made, and so I don’t think you have to get those diseases. I think they can be prevented….” (Nurse) [19] pp. 948
	“If you live better longer and don’t suffer from the infirmities associated with bad aging, like arthritis and diabetes and high blood pressure and cancer and Alzheimer’s, if you live without those illnesses, you’re going to feel better. You’re going to be more productive. Economically the country will benefit.” (Doctor) [19] pp. 951
	“[older people should not] wait for help to be given.” (Social worker) [17] pp. 2875
	“The key point is to accept changes… to perceive them as challenges not as loss.” (Occupational therapist) [17] pp. 2875

**Table 3:** Overarching theme- systemic context of attitudes to ageing

Theme subthemes	Illustrative quotation
(a) Resource shortfalls	“They are just very tangential…and I don’t have a ton of time for that. And I also sometimes don’t have patience for it.” (Resident) [20] pp. 479
(i) No time	“The work is so demanding. I have no time to ask questions of and talk to older people.” (Nurse) [23] pp. 63
	“I’d like to be able to talk to [older] people all day, but when you have seven other people to see, you don’t care if the little blue pill is this big or this shaped or what.” (Intern) [20] pp. 479
	“…I do not think that there are enough staff members here. Do we always treat the patients properly? No, we do not. Do I feel sorry about that? Yes, I do…We fail to do that due to our workloads. We do not have time. The number of nurses is quite low…” (Nurse) [15] pp. 182
(ii) No resources	“We can never find their relatives. We cannot ask them to purchase wet towels and diapers when required… We take these from other patients and use them. Sometimes, we end up purchasing them with our own money…” (Nurse) [15] pp. 182
	“senior care personnel is lacking, money is lacking… I cannot have positive aging without being able to afford good senior care…” (Psychologist) [17] pp. 2876
	“It’s hard when you don’t necessarily have a great plan for them when they leave hospital ….It’s a sense of disappointment in oneself and the system in not being able to provide a better outcome.” (Intern) [20] pp. 480
(iii) Inadequate training	“We could not identify what is wrong. We would just notice a change and express a concern. Because we haven’t got specific training in old age.” (Secure service nurse) [18] pp. 276
Opposing pole: well trained	“..most of us would have done some placement in elderly psychiatry as part of our training and ….feel competent to identify things like cognitive deficits.” (Secure service staff) [18] pp. 275
(b) Burnout and compassion fatigue	“I’m burning out. There’s a lot of sadness, there’s been a lot of goodbyes. When you work with the elderly, it doesn’t usually have a happy ending, you know? The goodbyes and uhm, dealing with poverty. Uh, it’s difficult and you seeing how much people suffer that are low income… so although I’d like to volunteer, I’d like to get away from that.” (Social Worker) [22] pp. 54
	“…we talk about compassion fatigue… after twenty-eight years of listening to client problems, caregiver problems, having been a caregiver myself… I’m tired.” (Geriatric community health worker) [22] pp. 54

#### Attitudes towards older persons

HCPs’ positive attitudes towards older persons included working with older people as rewarding [20, 21], that older people were to be revered and respected, exhibited resilience and strength, and had unique experience and knowledge [16, 22, 23] (Table 2). There was a recognition that a positive attitude towards older people depended on the practitioner’s mindset, i.e., considering deficiencies as challenges requiring adaptation rather than loss of function.

A contrasting theme was ‘Unwanted, dependent and difficult’. The subtheme ‘Unwanted and costly’ encompassed negative attitudes towards older people, including that they are a nuisance, attention seeking, cognitively impaired, and time-consuming (Table 2). The subtheme ‘Complex and challenging’ is especially related to older people residing in nursing homes. This complexity linked to multiple intersecting medical and social issues, lack of confidence of the practitioner [17], dislike of elder care, and therapeutic nihilism [20, 21]. Older people were described as difficult, because they did not follow recommendations [16]. However, this perspective was not universal, as other doctors felt that older people listened to them and respected their opinion [20]. An opposing pole of ‘Complex and challenging’ was ‘A welcome intellectual challenge’ for HCPs managing the complexity of issues of older adults.

The subtheme, ‘Dependent’, described dependence on the assistance of others and helplessness. Related to this dependency, some HCPs perceived that older people needed advocacy and person-centred care, because they were vulnerable and unable to communicate for themselves. The opposing pole of this theme was ‘Assertive and independent’.

#### Role of the older persons’ family

HCPs’ attitudes to ageing were shaped by the involvement or absence of the older person’s family (Table 2). ‘Meeting expectations’ of family including navigating family criticisms and priorities, which were not always aligned with the older persons’ needs [2] and could lead to deficient care [21].

The subtheme of ‘Abandonment’ referred to HCPs’ concern about families who appeared absent or abdicating responsibility, and also related to societal change.

The subtheme ‘Advocacy’ encompassed perceptions that absent family members enabled the gap between awareness of rights of the older person and enacting them in practise. To meet deficiencies in familial support, some HCPs described taking on an advocacy role for older patients [2].

#### Behaviour of health care professionals towards older persons

Healthcare professionals’ behaviours towards older people included undermining autonomy/self-determination, dehumanising mechanistic care, neglect, and differential care of older people compared to younger adults (Table 2). Paternalistic, disempowering behaviours were described, even violations of human rights (withholding treatment and neglect resulting in death), with older people sometimes invisible in care interactions. Differential care of older compared to younger people was described [2, 20, 22]. These behaviours contrasted with community nurses employed in health promotion, who adopted an empowerment approach, tradition, and folk wisdom, emphasising older people’s skills, experience, and abilities [23].

Older adults were considered unworthy of care by some HCPs, for example when poor health was considered self-inflicted. Older age was sometimes equated with pending death, contributing to a sense of futility justifying lack of care [20, 24].

#### Behaviour of older persons towards health care professionals

The core theme was ‘Challenging behaviours’ from older people towards HCPs (Table 2). Older people were described as abusive, dangerous, demanding, rejecting treatment, and dissatisfied. Some HCPs commented on the older person’s wish to please, which, in itself, would compromise their care. Older people’s invisibility was noted and attributed to their passivity, echoing earlier observations that those without family advocates are lost in healthcare systems. An opposing pole of this theme was ‘Respect for clinicians’ that older patients treated physicians in training with greater respect as professionals than younger patients.

#### Definition of an older person

Definitions of an older person varied, potentially influencing attitudes to ageing (Table 2). ‘More than chronological age’ recognised individual variation in ageing as more important than chronological age. ‘Illness and dependency’ conceptualised an older person as someone with multiple morbidities, chronic illness, dependency and loss of function, complexity, vulnerability to complications, and cognitive issues [15, 20, 24]. Some anti-ageing practitioners understood ageing as related to hormone deficits, deemed responsible for “inevitabilities of ageing” (fatigue, depression, muscle loss, and forgetfulness) [19]. Older patients were perceived by some as inherently at the end of life [20, 24]. The opposing pole of this subtheme was ‘Independent’.

‘Taking responsibility for ageing’ incorporated HCP views that ageing was largely self-determined and shaped by poor lifestyle choices [19]. The study of anti-ageing practitioners considered these undesirable health outcomes could be counteracted and the value of older people to society improved by anti-ageing measures and individuals taking personal responsibility [19]. This subtheme of taking responsibility for positive ageing was echoed elsewhere [17].

#### Overarching theme-systemic context of attitudes to ageing

Healthcare professionals explained their attitudes and behaviours towards older adults under two main themes; ‘Resource shortfalls’ and ‘Burnout and compassion fatigue’ (Table 3). Resource shortfalls included lack of time, healthcare staff, and system resources. Healthcare systems were described as inadequate to meet older peoples’ needs, including unsuccessfully providing medical solutions for social problems [20]. Training in older adult care and lack of equipment also influenced HCP behaviours [7, 15].

Healthcare professionals described burnout, compassion fatigue, and being traumatised working in a system with insufficient professional and social care. One participant uniquely mentioned their role as a carer in their personal life contributing to this emotional exhaustion [22].

### Secondary analysis-themes according to health care profession

A few studies compared the attitudes to ageing between different HCPs. Overall, themes were common across disciplines; however, emphasis varied in some professional groups.

#### Medical

Eight studies described perspectives of medical professionals regarding ageing [2, 14–20]. Commonality in emergent themes included negative perceptions of older age, often relating to multi-morbidity, perceived complexity, and time-consuming interactions, expected dependency [15–17, 20], and little likelihood of treatment success [15, 20].

Ageing was considered avoidable by anti-ageing practitioners (doctors formed the majority), and ‘bad ageing’—or the development of disease, depression and disability—as self-inflicted through lifestyle choices [19]. Doctors described less value in providing treatment for an older person compared to younger patients.

“It’s always a bigger save when you help a 35-year-old woman with 2 kids than it is to bring an altered 89 year old with a UTI back to her semi-altered state.” (Resident) [20] pp. 479

Therapeutic nihilism was prominently expressed by doctors [15, 20]. There was a sense of frustration, hopelessness, and demoralisation regarding providing care for older people in addition to the stress and resource use in managing complex needs.

“Treatment of older patients is considerably costly… You cannot get results from the treatment provided to them….” (Physician) [15] pp. 183

[describing work in a nursing home] “takes her joy of life away.” (Doctor) [14] pp. 43.

Doctors’ views regarding older adults were predominantly biological, focussing on multi-morbidity and treatment of disease, and conceiving the older patient’s social, emotional and physical needs as inconvenient, or not their role [15, 20]. Doctors focussed on their own role rather than being part of a multidisciplinary team (MDT).

“It gets frustrating when it feels like you’re doing a lot of social work but you’re not helping anybody… you’re not doing anything for them in the long term.” (Intern) [20] pp. 479

#### Nursing

Seven studies described nursing perspectives [14, 15, 18, 19, 21, 23, 24]. Nurses tended to focus on the emotional and physical burden of providing care for older people.

“…unpleasant things such as bathing an older person with deformations, changing the person’s diaper.” [14] pp. 43

The study of community nurses in Thailand explored characteristics of idealised healthy ageing [23].

“For me, they should not have any diseases. They must be totally healthy, both physically and psycho-logically.…. no difficulties with eating, chewing, or swallowing.” [23] pp. 60

There were parallels between the study of anti-ageing practitioners (largely doctors) [19] and the Thai nursing study [23]. Anti-ageing practitioners similarly viewed ageing as dualistic (good or bad), according to disease and disability burden, perceived as individually determined [19]. Similar nursing attitudes were reported regarding personal responsibility for illness, which linked to some older people being perceived as less worthy of care [21].

One nursing study uniquely described the important functions of older people within society more broadly:

“They should attend activities in the community ... They must work for the community and socialize within it. They should have a role in the community….support their neighbors.” [23] pp. 61

In contrast to doctors, nurses recognised the need for person-centred and holistic care of older people.

“Good nursing care involves meeting all the needs of the patient in terms of treatment and care, together with meeting his/her personal care needs.” [15] pp. 181

However, this was not universal, with a nursing home study describing dehumanised, task-orientated nursing care [24].

#### Social work

Four studies included social workers [2, 14, 17, 22]. Themes in this group were largely not distinct from other disciplines. A unique theme was that the healthcare system actively promotes regression in older adults through staff attitudes and time-pressure leading to inappropriate care and loss of dignity [14].

“When an older person enters the hospital, there is a certain approach towards them that makes them more dependent. The patient can be a very independent person… and somehow the attitude of the personnel toward them makes them change… they immediately put a diaper on people who did not need a diaper before….they don’t want to deal with it….there is no time.” [14] pp. 45.

#### Other allied health professionals

Occupational therapy and psychology were represented by one and three participants respectively, in one study [17], precluding comparison. Two allied health studies included personal representations of ageing, with fear the common sentiment [17, 22]:

“Sometimes I wonder when I will turn 80 and I will be ill…. Will there be people to take care of me or will there be a factory with robots to take care of me.” (Psychologist) [17] pp. 2876

“I don’t want to be aging….I don’t want to be old.” (Geriatric community health worker) [22] pp. 54

### Factors mediating attitudes to ageing

In addition to the systemic context of attitudes to ageing, relevant factors mediating HCP attitudes to ageing included gender [14, 16], whether the HCP chose to work solely with older adults or this population was simply one group within a broader age cohort of patients (i.e., self-selection into gerontological work) [14, 22], and cultural background [23].

Although HCP gender did not affect attitudes to old age when specifically evaluated [16], some differences in expression of perspectives were noted, with male physicians observed to be more judgemental and critical in their appraisal of older people and their families [14]. Female HCPs conceived good old age in terms of psychological health (self-acceptance) and social connections, whereas males referred to active lifestyle and positive thinking [16]. Physicians provided positive examples of older male patients and negative examples of women regarding adherence to treatment [16]. Older women were also depicted as having psychological problems hampering ageing [16].

Participants working in dedicated aged healthcare roles appeared to have more nuanced views of ageing and more empathetic responses to ageing. The contrast was especially evident comparing HCPs working in emergency contexts where older people were viewed as time-consuming, detracting from other work and a waste of resources [15] to community nurses promoting healthy ageing, who highlighted respect for older adults and facilitated autonomy and empowerment [23]. However, this apparent connection between positive attitudes and self-selection to aged-care employment was not universal, with striking negative behaviours and attitudes expressed by nursing and care staff of aged-care facilities [24].

Only one study specifically considered culture and spirituality as factors influencing attitudes to ageing [23]. Underlying Buddhist principles of helping others, generosity, being supported and supporting others, were highlighted as important to healthy ageing and utilised in practise by these community healthcare nurses. Further cultural links were made to the role of family in Buddhism [23, 26].

## Discussion

To our knowledge, this is the first qualitative systematic review examining HCPs’ attitudes to ageing. Despite the large number of initial citations identified through broad database searches, a limited number of qualitative studies were obtained. Of the 12 synthesised papers, nine were appraised as having the highest methodological quality. Five key themes regarding attitudes to ageing of HCPs emerged, encompassing predominantly negative HCP attitudes and behaviours towards older people, the role of family, behaviours of older adults towards HCPs, and definitions of an older person. Although less prominent, opposing perspectives reflecting more positive attitudes were found for many themes. An overarching theme of the systemic context for HCPs emerged, illuminating how resource shortfalls, compassion fatigue, and burnout affect attitudes and behaviours towards older people.

There were common themes across disciplines. However, doctors emphasised the complexity, multi-morbidity, and dependency of older adult patients, perceived as time-consuming and resource-intensive in a time-pressured healthcare system and whose care was often viewed as futile with recovery unlikely. The accompanying sense of therapeutic nihilism also influenced attitudes to older adult patients [14, 15, 20]. Notably, doctors did not seem to locate themselves within an MDT, considering social and physical care needs as an inconvenience assigned to other disciplines [20]. The studies of nursing perspectives focussed more on the burden of care, consistent with nursing roles as caregivers [15, 23, 24, 27]. Themes from social workers were largely consistent with the views of colleagues in other disciplines. Even within discipline groups, perspectives vary; for example, anti-ageing physicians hold particular attitudes to ageing, such as the belief that ageing is avoidable [19], which was not raised by other doctors. Considering there were relatively few studies for each HCP group, and some HCPs barely represented (such as psychologists), these preliminary findings may not be generalisable and require further exploration.

Previous literature reviews have used quantitative methods to examine HCPs’ attitudes to ageing [28–33]. The present qualitative review complements this quantitative work by elaborating how these attitudes have developed, providing contrast, context, and some explanations for these quantitative findings. Both positive and negative attitudes of nurses towards older adults have been previously reported [28–30], as in the present study, noting similar findings regarding the impact of demands of care provision on negative appraisals [29]. Tendencies to ignore older adult patients and instead speak to family members have been reported [29], echoing our findings of invisibility in HCP behaviours. Inconsistent quantitative findings are reported regarding the effects of gender, age, and education of nurses on attitudes to ageing [28]. Although there were some findings relevant to gender of both HCP (nursing or medical) and the older person in our review, age did not emerge as a factor influencing attitudes to ageing. Experience with and preference to work with older people have been reported as associated with positive attitudes in nurses [28, 29]. However, this was not consistently supported by our findings, especially in nursing home settings [24]. In line with our results, inadequate healthcare resources have been previously shown to influence negative nursing attitudes to older adult care [29], and adequate resourcing linked to positive attitudes [34]. Our identified lack of data regarding the influence of societal attitudes and cultural background of HCPs on attitudes of ageing has similarly been observed [28]. This needs further exploration, especially given findings from China, for example, where cultural traditions of inclusion of older people within family structures and as carers for younger generations (i.e., positive experiences of relationships with older people) were recognised as influencing nurses’ positive attitudes to ageing [35].

A review of the quantitative literature regarding doctors’ attitudes to ageing similarly demonstrated mixed findings, highlighting the importance of contextual factors, including more negative attitudes towards nursing home residents and the influence of healthcare systems [33]. Consistent with our findings, negative stereotypes of complexity, difficult behaviours, poor function, and disease were emphasised, alongside contrasting perspectives of older people as appreciative, pleasant, and contributors to society [33]. Healthcare systems, which are time-pressured, incentivise certain aspects of care, communicate poorly, and lack essential multidisciplinary staff, all increase burden on physicians, influencing their attitudes to older adults [33], echoed here. However, rather than valuing allied health staff and considering themselves part of an MDT providing holistic care, some doctors viewed broader health needs as inconvenient and less rewarding [2, 20]. Results varied as to whether training in geriatric medicine or exposure/experience working in this field influenced attitudes to ageing in physicians, although personal experiences with older people were somewhat protective against negative stereotyping [33]. In the present review, one study reported that junior doctors’ experience working with older adults improved attitudes and comfort managing older adults [20]. Similarly, there are inconsistent data on the effect of role models on physician attitudes to ageing [33] and this theme did not emerge in our review. Echoing our findings, the approach and milieu of healthcare systems was relevant to doctors’ attitudes to ageing [33]. Physicians were noted to have limited concepts of old age defined by chronological age [33], whereas our review highlighted variability and recognition of individual ageing across HCPs.

Aside from social work, we could not find quantitative reviews regarding the attitudes to ageing of other allied health professions. The one review exploring social workers’ attitudes towards older adults mostly comprised studies of students [32]. The single study which compared qualified geriatric and non-geriatric case workers found higher levels of death anxiety (fear of death of others and oneself) in the geriatric social workers and greater preference for working with the ‘frail elderly’ than younger clients [36]. However, whether this fear predated working with older adults or affected behaviours towards them were not examined. Non-geriatric case workers had a negative preference for working with older adults, reflecting the importance of HCP self-selection of work setting on attitudes to ageing.

The review highlighted that pressure within healthcare systems influences HCP attitudes towards older people. Lack of resources, including time, staff, and equipment [15, 20], lead to perceptions of older persons’ complex and often multiple needs as stressful, overwhelming, and onerous, and by extension that older people are unwanted and difficult [14, 15, 20, 21]. Resentment builds in HCPs when these complex needs cannot be met, subsequently projected on to the older person themselves and their families, and the latter sometimes perceived as absent or neglecting their duty [2, 15, 17]. These system pressures combine with an apparent absence of HCP mentors and positive role models and older adult specific training [18] identified in this review, fueling largely negative attitudes to ageing. It is therefore unsurprising that HCPs working in unsupported systems develop therapeutic nihilism and consider treatment futile [20, 21, 24]. Role models who convey respect, positive attitudes, and enthusiasm working with older people may helpfully shape the attitudes of more junior HCPs [37]. Similarly, HCP working clinically with older adults could deliver education to dispel negative myths about ageing and gerontological work [38]. Effects of education may be enhanced when combined with intergenerational contact [39]. Practical experience and work placements in aged-care settings may also improve attitudes to ageing [38]. The descriptions of under-resourced systems also suggest need for policy and advocacy to design and adequately fund systems of care [23].

Attachment and trauma-informed care may be useful lenses through which to consider relationships and attitudes of HCPs to older people, particularly noting the themes around relationships and burnout/compassion fatigue. Attachment theory holds that it is normal and not “regressive” to seek help at times of “sickness and calamity” [40]. Denial of this need represents a dismissing stance [41] versus a more secure state of mind that would value the other and normalise help and care, whilst those with more ambivalent states of mind are upregulated and anxious around their own and other’s needs for care [41, 42]. Different attachment states of mind tend to shape different approaches to interpersonal relationships, including clinical care [41], influencing the patient–clinician interaction in a bidirectional way [42]. The theme here of 'Unwanted, dependent and difficult' suggests a more dismissing stance, seeing dependence and needing care as intrinsically negative. Understanding what promotes the personal and systemic attitude of 'Rewarding and revered' will be important going forward.

Various factors influence empathy and engagement, potentially including a dismissing stance, the burden of care and compassion fatigue [5, 6], and the potential positive effects of culture [23]. Loss and trauma can notably disorganise or shift attachment state of mind [41]. Here, some HCPs’ experiences suggest traumatic overwhelm at providing care in under-resourced settings, along with “goodbyes”, implying deaths or other losses [22, 41]. Effects of compassion fatigue or burnout in clinical settings have been recognised [43], necessitating systemic and relational approaches to care of both patient and team, with supervision for both HCPs and team to sustain compassion in trauma-saturated settings [44, 45]. This procedural, relational, and systemic approach extends beyond didactic education around attitudes [44, 45].

A surprising omission in the reviewed studies was reference to the MDT, highly relevant to older adult healthcare. Healthcare professional groups largely spoke of their own roles in older adult care [20] without acknowledging the benefits of sharing patient care and responsibility within a team. Interprofessional learning—i.e., different disciplines learning with, from, and about each other to enhance collaborative care [46]—is an approach well suited to educating HCPs involved with older adults, where holistic care is essential to manage complex and varied needs. As this review highlights, attitudes to ageing may be shaped by various factors, including knowledge, workload, personal experiences, and expectations, perhaps differing between disciplines. The need for HCP training to improve understanding and attitudes to ageing is echoed elsewhere [7, 15]. Positive attitudes towards older people could be fostered by interprofessional collaboration and education [25].

This qualitative synthesis identified prominent negativity about older people with the focus on disease, dependence, disability, and difficult behaviours, and expressed therapeutic nihilism. These emergent themes may have been influenced by the inclusion of studies directly examining ageism [14, 20, 21], a well-recognised negative attitude towards older people [3]. This suggests a role for education and supervision, delivered in a trauma-informed and systemic way [44]. Supervision and Balint groups have been useful in other settings of challenge in clinician–patient relationship, with strong and often unconscious emotions [47, 48]. In mental health, training and supervision have been crucial aspects of trauma-informed care [45]. Balint groups, facilitated groups of HCPs, recognising the importance of emotional, personal and unconscious factors in clinical work [48], have been used in HCP training [47] and may be beneficial in older adult care.

## Limitations and strengths

The literature review was limited to peer-reviewed articles in English, with only a small number of studies identified, rendering saturation of themes unlikely. The restriction to the last 10 years provided a contemporary perspective, potentially at the expense of capturing more papers and diversity. The grey literature was excluded as this work has not been subject to peer review, validation, and scrutiny essential for quality assurance [49], but may have introduced publication bias [50]. The included papers were drawn from a range of settings and countries; however, the results may not be transferable. For example, there was only one study from an Asian country. This may be important given cultural nuances to concepts of ageing [28] and as the one Asian study [23] revealed important cultural factors influencing attitudes to ageing. In addition, study aims varied, with likely effects on derived themes. For example, those studies which directly explored ageism in HCPs [14, 20, 21] are likely to have identified more negative attitudes to ageing, consequently affecting emergent themes in the thematic synthesis. However, this was balanced using broad database search terms, which also identified papers on healthy ageing [17, 23].

Allied health professionals were under-represented, constituting a significant knowledge gap. Additionally, most of the studies, with some exceptions [2, 9], evaluated the self-report of HCPs without triangulation with the perspectives of the older people they care for. This may be important given observed theory–practise gaps [17] and as healthcare staff may present socially desired responses rather than revealing true personal perspectives [51]. In future research, navigation of various professional and systemic contexts may be aided by co-design and co-participation methodologies.

A strength of this analysis included minimising potential for reviewer bias by having three reviewers independently perform the quality ratings and qualitative analysis, with a fourth senior researcher reviewing results. Rigour of the analysis was further enhanced through reflexivity and discussion to reach consensus ratings.

## Conclusion

The systematic review highlights the breadth of HCP attitudes to ageing and how they may be shaped by professional experiences with older people and their families, and systemic factors. These attitudes may affect HCP responses to and care of the older person. Predominantly negative attitudes must be addressed to foster real and sustainable change in the care of older people. More study of different HCP discipline perspectives is needed to develop in-depth understanding, especially in allied health. Focus groups of MDTs may be a useful method of exploring interactions between HCP roles and attitudes to ageing. Future studies should also consider the role of culture and broader societal attitudes to ageing, as well as how personal experiences with older people may shape clinician attitudes. This review suggests that changing some of those attitudes might require more than simple education and work experience, as attitudes may be influenced by overwhelmed systems, secondary burnout, and vicarious traumatisation. Researchers, managers, and policy need to consider the local system and their relationship to attitudes and supports for clinicians, carers, and systems to understand what is needed to support sustainable change. The findings suggest that a relational and trauma-informed approach is needed.

## Data Availability

A data availability statement is not applicable for a systematic review.
